# Precursors of seizures due to specific spatial-temporal modifications of evolving large-scale epileptic brain networks

**DOI:** 10.1038/s41598-019-47092-w

**Published:** 2019-07-23

**Authors:** Thorsten Rings, Randi von Wrede, Klaus Lehnertz

**Affiliations:** 1 Department of Epileptology, University of Bonn, Venusberg-Campus 1, 53127 Bonn, Germany; 2 Helmholtz-Institute for Radiation and Nuclear Physics, University of Bonn, Nussallee 14–16, 53115 Bonn, Germany; 3 Interdisciplinary Center for Complex Systems, University of Bonn, Brühler Straße 7, 53175 Bonn, Germany

**Keywords:** Epilepsy, Complex networks

## Abstract

Knowing when, where, and how seizures are initiated in large-scale epileptic brain networks remains a widely unsolved problem. Seizure precursors – changes in brain dynamics predictive of an impending seizure – can now be identified well ahead of clinical manifestations, but either the seizure onset zone or remote brain areas are reported as network nodes from which seizure precursors emerge. We aimed to shed more light on the role of constituents of evolving epileptic networks that recurrently transit into and out of seizures. We constructed such networks from more than 3200 hours of continuous intracranial electroencephalograms recorded in 38 patients with medication refractory epilepsy. We succeeded in singling out predictive edges and predictive nodes. Their particular characteristics, namely edge weight respectively node centrality (a fundamental concept of network theory), from the pre-ictal periods of 78 out of 97 seizures differed significantly from the characteristics seen during inter-ictal periods. The vast majority of predictive nodes were connected by most of the predictive edges, but these nodes never played a central role in the evolving epileptic networks. Interestingly, predictive nodes were entirely associated with brain regions deemed unaffected by the focal epileptic process. We propose a network mechanism for a transition into the pre-seizure state, which puts into perspective the role of the seizure onset zone in this transition and highlights the necessity to reassess current concepts for seizure generation and seizure prevention.

## Introduction

Epilepsy is a neurological disorder that affects approximately 65 million people worldwide. It is intractable to anti-epileptic drugs in approximately one third of people with epilepsy^1^ and requires comprehensive care to address the adverse events of medical treatment, comorbid disorders, and quality of life issues^2,3^. Central to the burden for the person with epilepsy is the apparent unpredictability of seizures. Since the 1980s, the field of seizure prediction aims to predict the onset of a seizure well ahead of time to enable the person to take precautions against injury, and to open the door to novel, in time treatment to control the impending seizure^4,5^. A prospective trial of an ambulatory, brain-implantable seizure prediction device recently provided evidence that seizures are predictable, at least in some people with epilepsy^6^.

In addition to identifying seizure precursors with sensitivity and specificity sufficient for clinical applications, another important building block of a seizure prediction study is to identify the brain region(s) associated with the dynamics of such precursors^7^. Knowing how, when, and which brain region(s) are involved in the generation of a transitional pre-seizure state is of utmost relevance to improve our understanding of ictogenesis and, similarly, for delivering a locally confined, counteracting influence to prevent seizure generation. A number of previous studies on the predictability of focal onset seizures reported brain areas distant to the seizure onset zone to carry the relevant information^7–17^. This, at first glance counterintuitive observation contributed to the development of the concept of an epileptic network^18–23^, whose interactions extend well beyond the seizure onset zone over large regions of the brain. With this concept, seizures (even focal ones) and other related pathophysiological dynamics are regarded as emerging from, spreading via, and being terminated by network constituents (nodes and edges) that generate and sustain normal, physiological brain dynamics during the seizure-free interval.

**Figure 1:**
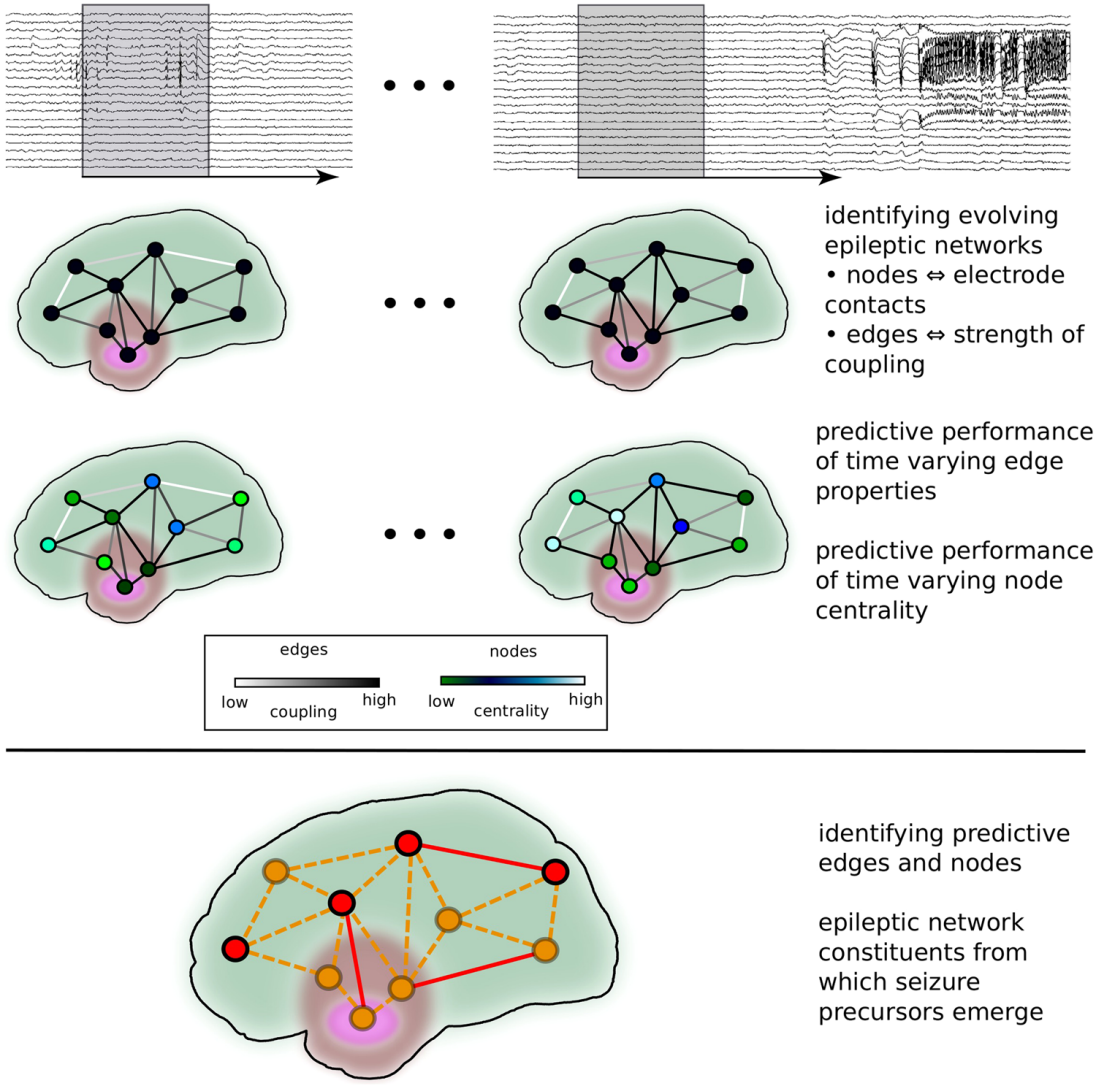
Identifying constituents of evolving epileptic networks from which seizure precursors emerge. The strength of coupling (level of synchrony) between pairs of sampled brain areas is estimated in a sliding-window fashion from multichannel iEEG data (Methods). In each window, electrode contacts are associated with nodes and the strength of coupling is associated with the weight of an edge between two nodes in the resulting snapshot network. From the temporal sequence of snapshot networks – evolving epileptic networks – the predictive performance of time varying properties of edges (weight) and nodes (centrality) is assessed using various downstream statistical analyses (Methods). Only if performance exceeds chance level, an edge resp. node is considered as predictive. Eventually, predictive edges and nodes (coloured red; non-predictive constituents are coloured orange) are identified and assigned to predefined functional modules (*S*: seizure onset zone; purple, *N*: neighbourhood; brownish, and *O*: other; greenish, Methods).

In most of the aforementioned studies on the predictability of focal onset seizures, identification of seizure precursors was achieved by characterising time-varying couplings (sometimes referred to as connectivity) between pairs of brain regions from long-lasting (mostly intracranial) EEG recordings. In the context of a functional brain network^24^, which includes the epileptic network^23^, these couplings represent edges that connect nodes which represent brain regions. In previous concepts, nodes connected by an edge carrying predictive information were assumed to be involved in the generation of seizure precursors. Here we aimed to shed more light on the role of these and the other nodes in an evolving epileptic network, whose edges vary in time and that recurrently transits into and out of seizures. To this end, we employ a statistical approach^4,7,8^ to identify seizure precursors from time-varying changes of properties of edges and nodes. For the latter, we use the fundamental concept of centrality^25^ to assess the predictive role of each node in an evolving epileptic network (Fig. 1; Methods).

We find that nodes connected by an edge that carries predictive information are – unexpectedly – not the most central ones in an evolving epileptic network, their time-varying changes of centrality, however, also carry information heralding an epileptic seizure. Interestingly, despite the evidence for these network nodes to be involved in the generation of seizure precursors, they are associated with brain regions deemed unaffected by the focal epileptic process.

## Results

43 patients with epilepsy at the Department of Epileptology of the University of Bonn that were part of previous studies^7,26^ were included in this retrospective study. Pre-surgical invasive evaluation with chronically implanted intracranial electrodes captured a total of 249 clinical seizures. For our investigations, we only considered clinical seizures that met our selection criterion (Methods). With this criterion the number of patients was reduced to 38 and the number of seizures to 97 (range 1–7). Intracranial EEG (iEEG) recordings with, on average, 56 electrodes (range 14–120) lasted, on average, 3.5 days (range 0.8–9.5). Table 1 shows demographic information for these patients.

**Table 1:** Patient demographics.

Patient	Age	Sex	Dur	N_szr_	D_tot_	D_int_	D_pre_	E_tot_	E_S_	E_N_	E_O_	P_e_	P_nb_	P_ns_
1	54	Male	46	1	228	224	4	86	3	2	81	220	0	3
2	34	Male	29	7	111	85	26	26	5	5	16	27	5	10
3	15	Female	10	4	162	146	16	66	44	0	22	91	3	7
4	45	Female	42	1	146	142	4	48	12	2	34	37	0	4
5	25	Female	21	1	82	78	4	58	10	1	47	3	0	0
6	22	Male	23	5	94	74	20	74	10	1	63	353	10	0
7	57	Male	51	3	71	61	10	72	14	11	47	0	12	5
8	39	Female	11	3	91	79	12	52	11	3	38	342	0	0
9	24	Female	23	2	20	14	6	42	20	0	22	28	1	5
10	34	Male	33	4	70	54	16	52	20	4	28	14	11	6
11	25	Male	24	3	26	17	9	58	3	5	50	0	15	23
12	43	Female	27	3	94	85	9	56	8	0	48	0	3	4
13	29	Male	17	4	92	76	16	120	20	4	96	125	4	19
14	38	Male	15	2	52	44	8	46	8	4	34	0	2	2
15	44	Female	31	1	103	99	4	14	4	0	10	14	2	1
16	52	Male	52	1	49	45	4	42	5	4	33	4	1	1
17	45	Male	24	3	116	107	9	72	28	0	44	292	10	15
18	31	Female	14	2	74	69	5	36	11	1	24	18	0	2
19	25	Female	6	5	161	142	19	90	8	1	81	44	5	11
20	53	Female	13	1	46	42	4	24	11	3	10	43	1	1
21	62	Female	50	3	94	84	10	56	39	1	16	0	6	2
22	44	Female	30	3	129	117	12	46	30	0	16	0	1	3
23	25	Male	13	3	18	8	10	30	5	4	21	56	2	0
24	26	Female	10	1	26	22	4	16	5	4	7	26	0	1
25	54	Female	49	1	67	63	4	62	9	7	46	83	1	2
26	27	Female	16	2	163	155	8	48	10	2	36	421	7	9
27	28	Female	25	2	126	121	5	46	21	1	24	639	2	0
28	19	Male	9	2	47	40	7	78	34	2	42	0	1	12
29	26	Female	18	3	97	85	12	36	10	0	26	9	1	6
30	37	Male	5	2	103	95	8	46	10	4	32	262	2	11
31	25	Male	26	2	32	25	7	78	10	0	68	107	4	18
32	37	Male	2	4	68	52	16	65	6	0	59	23	2	3
33	15	Female	11	2	36	28	8	30	8	7	15	7	1	1
34	24	Male	4	2	67	59	8	65	6	3	56	80	6	1
35	22	Male	18	3	19	7	12	38	4	2	32	2	0	3
36	29	Female	12	2	37	29	8	88	6	1	81	86	2	9
37	41	Female	13	2	127	119	8	118	13	5	100	107	2	5
38	27	Female	13	2	67	59	8	30	6	7	17	20	1	5
$\varnothing$	34		22	3	85	75	9	56	13	3	40	94	3	6
Σ				97	3211	2851	360					3583	126	210

Dur = Duration of epilepsy in years; N_szr_ = number of seizures; D_tot_ = total recording duration in hours; D_int_ = total duration of inter-ictal periods in hours; D_pre_ = total duration of pre-ictal periods in hours; E_tot_ = total number of recording sites; E_S_ = number of recording sites in functional module *S*; E_N_ = number of recording sites in functional module *N*; E_O_ = number of recording sites in functional module *O*; P_e_ = number of predictive edges; P_nb_ = number of predictive nodes based on betweenness centrality.

*C*^*B*^; P_ns_ = number of predictive nodes based on strength centrality *C*^*S*^. See Methods for definition of functional modules and explanation of employed centrality concepts.

The study was approved by the ethics committee of the University of Bonn, and all patients had signed informed consent that their clinical data might be used and published for research purposes. A parent or the nominated legal carer gave written informed consent on behalf of the participant if below the age of 18. All experiments were performed in accordance with relevant guidelines and regulations.

### Which nodes are connected by a predictive edge?

**Figure 2:**
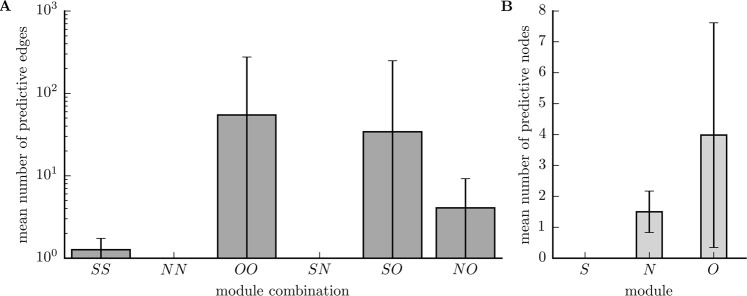
Mean numbers of predictive edges and nodes grouped by functional module. Bar graph of the (mean ± standard deviation) number of predictive edges (**A**) and nodes (**B**) per patient (pre-ictal periods of 78 seizures from 38 patients). Predictive edges connect (predictive and non-predictive) network nodes (brain regions) within and between functional modules (*S* SOZ, *N* neighbourhood, *O* other). Note that there may be more than one predictive edge and more than one predictive node per pre-ictal period and these edges may connect different nodes. Predictivity of nodes estimated with strength centrality.

In 31 out of 38 patients (81.6%), we identified a total of 3583 predictive edges (Methods; on average, 7.8% of all edges, range: 0.2–61.7%; Table 1) that carried information predictive of an impending seizure (78 out of 97 seizures). Predictive edges most often connected network nodes in brain regions not affected by the focal epileptic process (other, functional module *O*), followed by connections between nodes in the seizure onset zone (SOZ, functional module *S*) and nodes in non-affected regions (module *O*) and by connections between nodes located near the SOZ (neighbourhood, functional module *N*) and in module *O* (Fig. 2A). There were only a few predictive edges within module *S* or between modules *S* and *N*. These results corroborate previous findings^7^.

### Are nodes connected by a predictive edge the most central ones?

**Figure 3:**
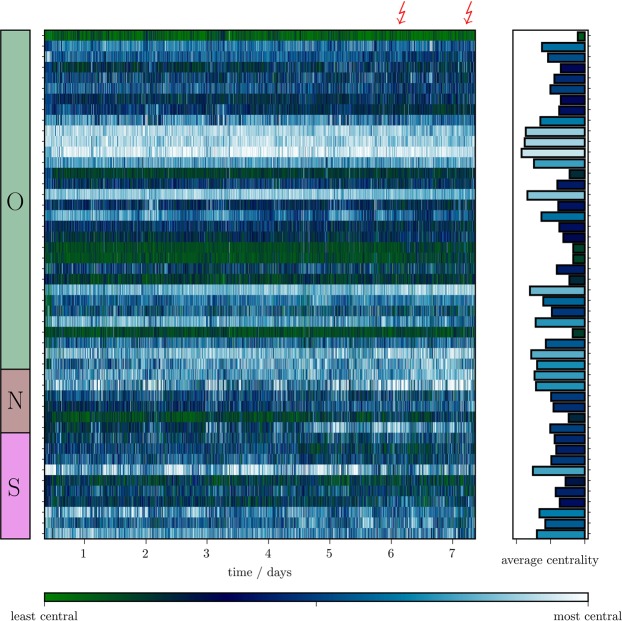
Time-varying changes of node centrality in an epileptic network. Exemplary time course of centrality of each node in a patient’s evolving epileptic network derived from multichannel iEEG recorded continuously over more than seven days. Data grouped by functional module (*O* other, *N* neighbourhood, *S*  SOZ). Bolts on top of the plot mark times of seizure onset, and tics on x-axis denote midnight. On average, the most important node belongs to functional module *O*.

Having identified pairs of nodes that are connected by a predictive edge, we next investigated the role of these and the other nodes in an evolving epileptic network. To this end, we estimated the centrality of nodes in each snapshot network with strength centrality and with betweenness centrality (Methods; Fig. 3). Despite their conceptual differences (Methods), both centrality concepts mostly led to qualitatively comparable results. If not stated otherwise, in the following we present our findings obtained with strength centrality.

We denote a pair of nodes connected by a predictive edge with (n_*h*_, n_*l*_), with node n_*h*_ being more central than its partner n_*l*_, and determined their rank relative to the centrality values of the other nodes in each snapshot network. During pre-ictal periods, n_*h*_ nodes ranked, on average, among the most central one-third of nodes in each pre-ictal snapshot network, while n_*l*_ nodes ranked slightly above the least central one-third. The variability of the mean relative rank seen for n_*h*_ and n_*l*_ nodes, however, was quite high (n_*h*_: 0.15–0.99; n_*l*_: 0.05–0.90), and in none of the cases could we observe either of these nodes to be the most central one during all of the pre-ictal periods. Despite the pronounced spatial and temporal variability of their centrality, (n_*h*_, n_*l*_) nodes were linked over a long period to specific functional modules or combinations thereof. Highest percentage linkage times were seen for modules *O* (85.5%) and *S* (86.0%), followed by the module combinations *NO* (n_*h*_ is linked to *N* and n_*l*_ is linked to *O* for 67.4% of the time; 100% minus this value holds for the reverse linkage), *SN* (52.8%), and *SO* (56.0%).

Interestingly, if we consider the most central node (maximum centrality value) in each pre-ictal snapshot network, we observed this node to be linked to functional module *O* during about two-thirds of the pre-ictal period during almost one-third to functional module *S*, and only rarely to functional module *N* (<10%).

Of note, characteristics of (n_*h*_, n_*l*_) nodes during inter-ictal periods were comparable to those seen during pre-ictal periods. These findings are quite unexpected if we consider the following: given our methodologies, an edge carries predictive information if its time-varying weights during pre-ictal periods differ significantly from those during inter-ictal periods (using the mean phase coherence as an estimator for that weight). Since edges in each of our evolving weighted epileptic networks are associated with time-varying, pair-wise estimates of strength of coupling and since time-varying centrality indices are also derived from these estimates, we would have expected to also observe differences (possibly less pronounced) between the distributions of pre-ictal and inter-ictal centrality values of (n_*h*_, n_*l*_) nodes – or at least of one of them – connected by a predictive edge. The discrepancy as well as the comparably stable centrality values for both these nodes can be explained by a spatial reordering of their centrality when epileptic networks transit from the inter-ictal to the pre-ictal period.

### Are temporal changes in node centrality predictive of an impending seizure?

The aforementioned spatial reordering indicates that temporal changes of node centrality carry information predictive of an impending seizure. In order to test whether this is indeed the case, we applied our statistical approach (Methods) to identify predictive nodes. We identified a total of 210 predictive nodes (on average, 6 nodes per patient; 9.9% of all nodes, range: 1.5–39.7%) in 33 patients (84.6%) and prior to 61 of 97 seizures. Predictive nodes were confined to functional modules *O* (other) and *N* (neighbourhood), and we could not observe any predictive nodes related to the SOZ (module *S*; Fig. 2B).

Interestingly, these predictive nodes played no central role in evolving epileptic networks, neither during pre-ictal nor during inter-ictal periods (median relative rank of nodes during pre-ictal period: 0.57 and during inter-ictal period: 0.56).

### Do predictive edges connect predictive nodes?

Having identified both, predictive edges and predictive nodes along with their region- and timescale-specific characteristics eventually enabled us to address the main point of our investigations, for which we considered the following cases:

- both nodes connected by a predictive edge carry predictive information (c1);
- one of the nodes connected by a predictive edge carries predictive information (c2);
- nodes connected by a predictive edge do not carry predictive information, however, there is at least one predictive node nearby (c3);
- nodes connected by a predictive edge do not carry predictive information and predictive nodes are farther away (e.g., different lobe or contralateral; c4);
- there are predictive edges only (c5).

Note that a node can contribute more than once to each of the aforementioned cases and more than once to different cases. Here, we did not consider the case of solitary predictive nodes, which we observed in 7 patients: 51 solitary predictive nodes represented about one-quarter of all predictive nodes (Table 1). 90% of these solitary predictive nodes were located in module *O*; the remaining nodes were located in *N*. None of these nodes played a central role in evolving epileptic networks, neither during pre-ictal nor during inter-ictal periods.

While cases c1 and c2 are the most intuitive ones, with c3 we take into account the dense spatial sampling with intracranial electrodes as well as different intracranial electrodes targeting the same brain region. Case c4 takes into account predictive edges that are spatially unrelated to predictive nodes (e.g. different lobes or contralateral hemisphere), while case c5 considers the observation of only predictive edges and is listed for control.

**Figure 4:**
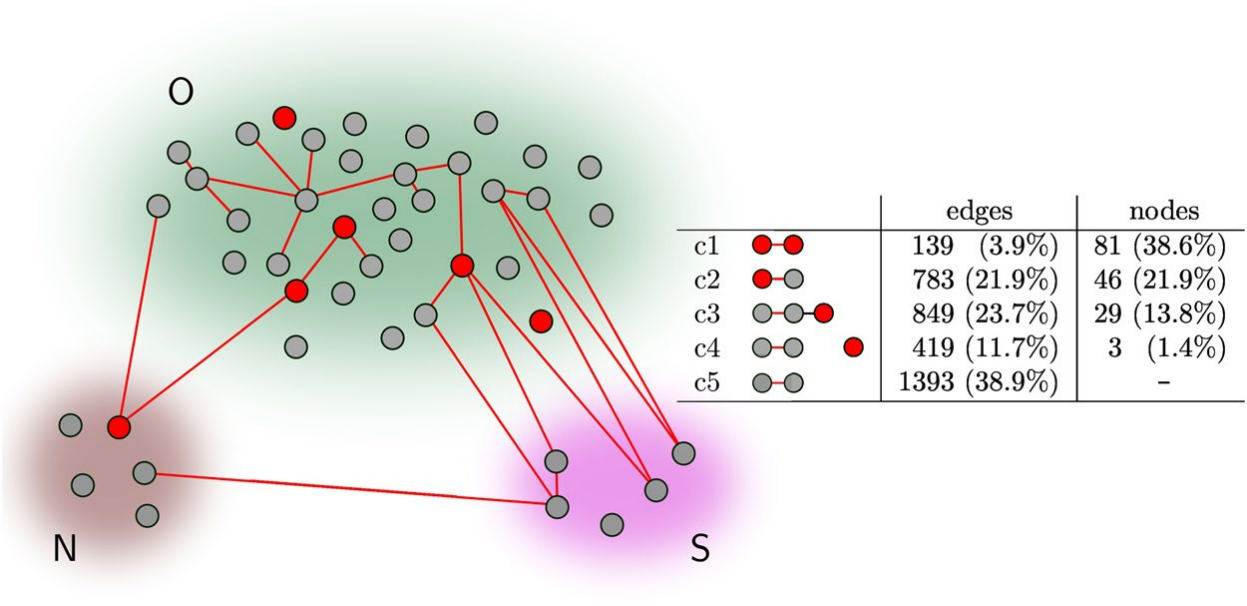
Predictive edges and predictive nodes. Schematics summarizing our findings of the spatial distribution of predictive edges connecting predictive (red) and/or non-predictive nodes (grey) within and between functional modules (*O* other, *N* neighbourhood, *S* SOZ). For the sake of clarity, we do not show non-predictive edges. The table reports the number of network constituents contributing to each case (c1–c5). Percentages refer to the total amount of the respective predictive network constituent. We do not report the number of solitary predictive nodes (24.3% of all predictive nodes).

Summarizing the cases c1, c2, and c3, about half of predictive edges connected three-quarters of predictive nodes, and these findings, highlighted in Fig. 4, allow us to conclude that predictive edges indeed connect predictive nodes in the majority of cases. The associated brain regions, despite being involved in the generation of seizure precursors, appear to play only a secondary role in the evolving epileptic network’s global dynamics and correspond to areas far off the seizure onset zone, thus usually being deemed unaffected by the focal epileptic process. The other half of predictive edges connected non-predictive nodes (cases c4 and c5) most often from modules *S* and *O* as well as from within functional module *O*. The centrality ranking of these nodes compared to the one seen for predictive nodes during both the pre-ictal and inter-ictal periods.

Of note, both predictive nodes and edges yielded redundant information of an upcoming seizure in 49 out of 97 seizures. For another 12 seizures (for which we could not observe predictive edges), predictive nodes provided non-redundant information. On the patient-level, predictive nodes provided non-redundant information in 5 patients.

### Which network modifications constitute a pre-seizure state?

**Figure 5:**
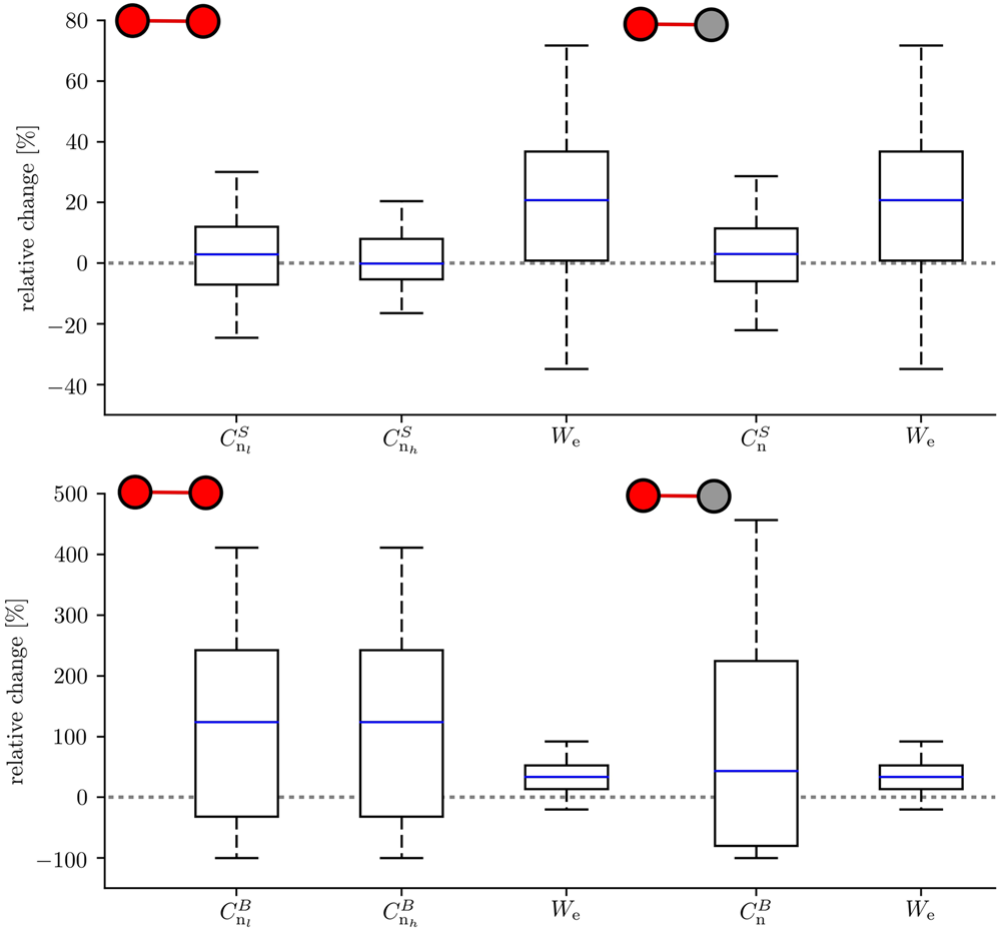
Distributions of pre-seizure changes in characteristics of predictive edges and nodes. Boxplots of the relative change *δ* in weights *W*_e_ of predictive edges connecting predictive nodes and in centrality values (top, strength centrality *C*^*S*^) and (bottom, betweenness centrality *C*^*B*^) of predictive nodes connected by predictive edges (left: both nodes (n_*h*_, n_*l*_) carry predictive information; case c1; right: only one node (n) carries predictive information; case c2). Relative changes are calculated as *δ* = (*M*_p_ − *M*_i_)/*M*_i_, were *M*_p_ and *M*_i_ denote placeholders for the medians of the respective characteristics from the pre-ictal and inter-ictal periods. Bottom and top of a box are the first and third quartiles, and the (blue) band inside a box is the median of the distribution. The ends of the whiskers represent the interquartile range of the data. Note that the medians of relative change in edge weights for cases c1 and c2 differ only by 0.9% for *C*^*S*^ and by 10.5% for *C*^*B*^.

Knowing that predictive edges connect predictive nodes in the majority of cases, we now aim at a possible network mechanism for the generation of seizure precursors. To this end, we investigate which alterations of characteristics of predictive constituents accompany the epileptic network’s transition from the inter-ictal to the pre-ictal state. We find the edge weights to undergo, on average, a pronounced pre-ictal increase that exceeds the inter-ictal level by about 20% (Fig. 5). At the same token, the node strength centrality values increase only moderately (by about 3%), in contrast to their betweenness centrality values which undergo a pronounced pre-ictal increase (by about 50%).

The observed change of weights of predictive edges reduces the length of paths passing through the nodes connected by these edges. The fact that the nodes’ strength centrality values vary only weakly points to a reduced weight of non-predictive edges connecting these nodes with the other nodes in the network. This balancing of edge weights together with an increased betweenness centrality of predictive nodes indexes a large-scale rearrangement of shortest paths, which affects most strongly specific network constituents. The latter renders the associated brain regions and connections between them bottlenecks in the evolving epileptic network and includes them in the generation of seizure precursors.

## Discussion

The time-resolved estimation of interactions between pairs of brain regions from recordings of their gross electrical activities has been repeatedly shown to allow reliable identification of seizure precursors, with lead times in the order of several tens of minutes to few hours^4,5^. In the context of an epileptic network^19–22,27^, that evolves in time, such estimation is equivalent to estimating the time-varying weight of network edges that connect network nodes representing brain regions^23^. However, interpreting the role of nodes – connected by an edge that carries predictive information – in seizure generation is not straightforward. It is hypothesized that the associated brain regions may be involved in the generation of seizure precursors, thus representing targets for therapeutic interventions that aim at preventing seizure generation^28–30^. Investigating multi-day, multi-channel iEEG data capturing almost 100 seizures from 38 epilepsy patients, we here tested this hypothesis and quantified the role of individual nodes in each individual evolving large-scale epileptic network with centrality, one of the most fundamental concepts in network science.

In contrast to what one would expect intuitively, our findings indicate that nodes identified as most central for the evolving epileptic network are not connected by predictive edges (i.e., edges that carry information predictive of an impending seizure). Investigating time-varying changes of node centrality, we observed – to our knowledge for the first time – that these changes also carry predictive information. Interestingly, the vast majority of such predictive nodes were connected by most of the predictive edges, but predictive nodes never ranked among the most central ones. Importantly, these network nodes correspond to brain areas far off the seizure onset zone (SOZ), such as different ipsilateral lobes or regions from the contralateral hemisphere, which are usually deemed unaffected by the focal epileptic process. Our findings thus put into perspective the role of the SOZ in seizure generation and highlight the necessity to reassess current concepts for seizure generation and seizure prevention.

### Revisiting the role of the SOZ in seizure generation

Identifying the seizure onset zone (or seizure onset area) is the current gold standard for an identification of the epileptogenic zone, defined as the brain area indispensable for seizure generation and whose removal should stop seizures. The SOZ is usually referred to as the “area of the cortex from which clinical seizure are (actually) generated”^31^ or as the “area of cortex that initiates clinical seizures”^32^, among others. It is determined primarily by identifying the (mostly invasive) EEG electrode(s) with the earliest onset of seizure activity.

Notwithstanding the high relevance of identifying the SOZ for the presurgical evaluation of candidates for epilepsy surgery, terms such as “generate”, “initiate”, or “originate” implicitly attribute an active seizure-precursor-mediating role to the SOZ, and such an attribution underlies the vast majority of seizure prediction studies, studies on brain stimulation^29,33–35^ and on modelling seizure dynamics^36^.

Our findings indicate that the SOZ does not generate seizures. In contrast, they highlight the high relevance of brain outside of the SOZ in generating seizure precursors, which points to a neuromodulatory input to the SOZ that permits or pushes the SOZ to seize. This input originates from brain regions that are part of the physical and physiological substrate from which seizures arise and spread – the epileptic network –, even if these brain regions do not directly participate in the electrographic seizure activity^4,5,17,26,37–39^.

### Revisiting the importance of the SOZ in evolving epileptic networks

A number of previous studies on EEG-derived epileptic networks reported most important nodes – identified with various centrality indices – to coincide with the SOZ. These nodes have been interpreted as so-called network hubs and were assigned a leading role in seizure generation^40–46^. Most of these studies, however, investigated only a limited number of brain regions and only a few pathologic states (e.g. during seizure onset or during inter-ictal epileptiform discharges). A more recent study investigated node importance (based on the centrality indices also employed here) in evolving large-scale epileptic networks. These networks were derived from multi-channel, continuous multi-day iEEG recordings that covered multiple lobes from both brain hemispheres of 17 epilepsy patients and that captured a large spectrum of various pathophysiologic and physiologic processes, acting on different timescales^47^. Strength centrality indexed the SOZ and betweenness centrality indexed brain regions far off the SOZ as most important most of the time. However, the high interindividual variability^48^ together with the strong fluctuations of highest importance over time – seen with both centrality indices – impeded on drawing clear-cut conclusions about the most important brain region in evolving epileptic brain networks. Our results corroborate these findings to a large extent; with both centrality indices we observed the most central node to be confined to brain regions far off the SOZ for most of the time and to the same extent for inter-ictal and pre-ictal periods. Although this node was not connected to an edge that carried information predictive of an impending seizure, it was nonetheless functionally related to network constituents (nodes and edges) being involved in the generation of seizure precursors. Future studies that aim to shed more light into this mismatch should also consider numerical inaccuracies when identifying evolving epileptic networks from noisy iEEG signals and estimating centralities as well as the notoriously difficult problem of ranking in complex networks^49^.

### A proposal for ictogenesis in evolving epileptic networks

**Figure 6:**
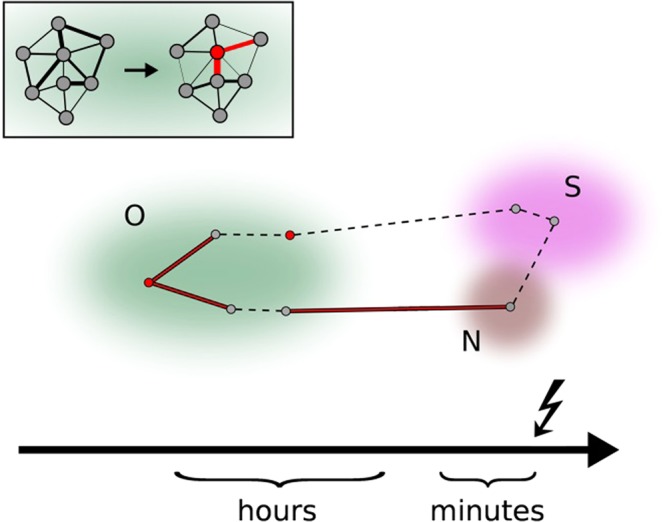
Ictogenesis in evolving epileptic networks. Schematics on how, when, and from which brain regions seizure precursors are being generated in evolving large-scale epileptic networks. Predictive edges connecting predictive (red) and/or non-predictive nodes (grey) within and between functional modules *O* (other; greenish), *N* (neighbourhood; brownish), and *S* (SOZ; purple). Non-predictive edges are shown as black dotted lines. The inset exemplifies the rearrangement of the epileptic network’s path structure that results in a formation of a bottleneck.

Our findings together with those accomplished in previous retrospective EEG-based seizure prediction studies^4,5,50^ allow us to formulate the following scenario on how, when, and where seizure precursors are being generated in evolving large-scale epileptic networks (Fig. 6).

Ictogenesis is induced by a rearrangement of the epileptic network’s path structure that is possibly triggered by endogenous and/or exogenous factors and that results in a formation of bottlenecks. Earliest indications for such a critical formation – with lead times ranging between several tens of minutes up to hours – can be observed when characterising functional interactions (or edges) within and between brain regions far off the seizure onset zone (SOZ), i.e., different lobes and regions from the contralateral brain hemisphere (functional module *O*). Likewise, comparable predictive information can be achieved with characterising the time-varying centrality of associated network nodes. These brain regions are usually deemed unaffected by the focal epileptic process, and as part of the large-scale epileptic network they generate and sustain normal, physiological brain dynamics during inter-ictal periods. Seizure precursors with long lead times might thus coincide with Gowers’ prodromes^51^, and the high spatial variability of precursor occurrences might explain the high intra- and interindividual diversity of prodromes. We expect that progress in characterising time-varying aspects of involved network constituents can help to further improve our understanding of mechanisms underlying the emergence of these early seizure precursors.

Subsequent indications for ictogenesis – with lead times in the order of a few minutes – can eventually be observed near or within the SOZ with analysis techniques that characterize specific (linear and/or nonlinear) aspects of the (patho-)physiological dynamics of only these brain regions, and not taking into account network-wide interactions. Given their close (spatial and temporal) proximity to the ictal event, seizure precursors from functional modules *N* and *S* are often thought of as being more specific for ictogenesis. When taking into account network-wide interactions, however, these precursors appear to result from functional interactions (edges that carry predictive information) with network nodes at which the ictogenic process started long before. We therefore hypothesise that late seizure precursors merely represent a time-delayed, ictogenesis-reflecting epiphenomenon. It should be noted though that a verification of this hypothesis requires identification of causal relationships. We expect further insights from recent developments that aim at characterising weighted and directed interactions in complex systems such as evolving large-scale epileptic brain networks^17,26,52^.

### Prospects on controlling ictogenesis in evolving epileptic networks

Current neuromodulatory epilepsy therapies either build upon a spatially targeted stimulation at the presumed site of seizure generation (SOZ) or at network hubs (SOZ or thalamus) or upon an unspecific and diffuse brain stimulation (e.g., via (invasive or transcutaneous) stimulation of the vagal nerve). Devices are designed to either stimulate constantly or periodically or to stimulate during the ictal phase (which builds upon an early and reliable seizure detection^53)^, and none of these devise have yet been coupled to seizure prediction systems. Our findings indicate that neither the timing nor the targeted spatial locations of current neuromodulatory epilepsy therapies can be accepted as suitable for reliably controlling ictogenesis in evolving epileptic networks. This might also explain the comparatively limited success of these therapies. We hypothesise that control techniques that aim at better targeting the spatial and temporal emergence of early seizure precursors^54,55^ combined with novel approaches to track changes in resilience of evolving epileptic networks^56^ as promising avenues for further research.

### Limitations of the study

Our retrospective study was based on electroencephalographic data recorded intracranially during the presurgical evaluation, and a number of variables (such as transient effects of surgery, sleep deprivation, medication tapering, or multi-day rhythms^57,58^) could confound the delineation of pre-ictal from inter-ictal periods. Moreover, we chose a statistical analysis design and compared the distributions of qualifiers (mean phase coherence for edges; centralities for nodes) from the inter-ictal with those from the assumed pre-ictal period. We therefore chose to not report on characteristics of seizure prediction performance (such as sensitivity, specificity, prediction times, or the portion of time under false warning)^4^.

We adopted the SOZ that was determined at the time of the presurgical evaluation; however, the limited coverage of brain with intracranial electrodes inherently precludes an exact delineation of the margins of the SOZ. By the same token, the limited coverage hampers the sampling of an evolving epileptic network with sufficient spatial and temporal resolution^5^, which calls for improvements in intracranial recording technology^38^.

Our study revealed that predictive nodes are not the most central nodes in an evolving epileptic network, but we cannot yet make a similar statement for predictive edges. Recent modifications of centrality concepts for nodes to those for edges^59^ are expected to provide further insights into the role of network edges and nodes in ictogenesis.

## Methods

### Data

Our investigations are based on patient-specific evolving epileptic networks that we derived from intracranial electroencephalograms (iEEG) recorded continuously for a prolonged period (typically several days) from chronically implanted depth electrodes and subdural grid- and/or strip-electrodes as part of the pre-surgical evaluation of intractable epilepsies (Fig. 1). Depth electrodes were equipped with 10 or 8 cylindrical contacts of length 2.5 mm and an intercontact distance of 4 mm. Strip electrodes consisted of 4 or 8 contacts with an intercontact distance of 10 mm, and grid electrodes had 8 × 4 or 8 × 8 contacts with an intercontact distance of 10 mm. Data were band-pass-filtered between 1–45 Hz, sampled at 200 Hz (sampling interval 5 ms) using a 16 bit analogue-to-digital converter, and referenced against the average of two electrode contacts outside the presumed focal region. Reference contacts were chosen individually for each patient, and their data was disregarded in this study.

Since number and anatomical locations of intracranial electrodes were adapted to the patients’ needs and were thus highly non-uniform (Table 1), we assigned electrode contacts to functional modules^7^. Module *S* comprised contacts where first ictal discharges were recorded (seizure onset zone (SOZ)^31^; about 23% of all contacts) and module *N* (neighbourhood; about 6% of all contacts) those contacts not more than two contacts distant to those from module *S*. All remaining contacts were assigned to module *O* (other; about 71% of all contacts, with more than half of these contacts sampling the contralateral hemisphere).

### Identifying evolving epileptic networks

Here we followed previous studies^47,48,60–62^ and identified evolving epileptic networks from iEEG signals by associating network nodes with electrode contacts and the weight of network edges with the time-varying strength of coupling between pairs of sampled brain regions, regardless of their anatomical connectivity. For the latter, we employed an established method for investigating time-variant changes in phase synchronization from brain signals (mean phase coherence *R*)^63^, particularly since this method has been repeatedly shown to reliably identify seizure precursors^7,8,11,13,14,17,64^. For our investigations, we moved a sliding window along the iEEG, and inside each window (duration 20.48 s; corresponding to 4096 data points), we computed *R* in a frequency-adaptive manner^65^ between each pair of sampled brain regions. *R* takes on values between 0 and 1 indicating either complete asynchrony or complete synchrony.

Having calculated *R* for all pairs of brain regions, we derived – for each window – a synchronisation matrix whose non-diagonal elements were associated with the adjacency matrix. This matrix represents an undirected, weighted snapshot network (Fig. 1). In the adjacency matrix, we set the diagonal elements to zero in order to avoid self-loops. In addition, we divided each matrix element by the mean strength of coupling to account for a possible influence of the latter^66^. With these steps of analysis, we derived a temporally highly resolved sequence of snapshot networks (evolving epileptic networks) spanning several days for each patient.

### Estimating time-varying centrality of nodes in evolving epileptic networks

**Figure 7:**
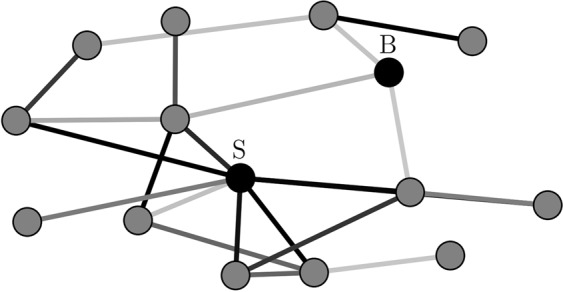
Different centrality indices identify different nodes as most central. Exemplary weighted network consisting of 15 nodes. Edge weights are colour coded with darker colours representing larger weights. “B” and “S” mark node identified as most central with betweenness centrality and strength centrality, respectively.

The role of individual nodes in a network can be assessed with the concept of centrality^25^. This concept allows for various interpretations, which is reflected in a number of centrality indices. Here, we characterised a node’s centrality with strength centrality *C*^*S*^ and with betweenness centrality *C*^*B*^ since these indices provide complementary information about the role of a node in functional brain networks^47,48,67^ (Fig. 7). Strength centrality *C*^*S*^ assumes the highest value for a node having the highest sum of weights of edges incident on that node. A node with a high *C*^*S*^ is central since it interacts strongly with many other nodes in the network. Betweenness centrality *C*^*B*^ assumes the highest value for a node that lies on the largest number of shortest paths between other pairs of nodes. We here related the “length” of a path between two nodes to the sum of the inverse weights of edges along this path^68^. A node with a high *C*^*B*^ is central since it connects different regions of the network by acting as a bridge and thus can affect the information flow in the network.

We estimated both *C*^*S*^ and *C*^*B*^ for each node (electrode contact) in each snapshot network in the temporal sequence of epileptic networks (Fig. 1) and rank the respective centrality values in an ascending order to identify the most central node.

### A statistical approach to identify predictive edges and nodes

We employed a statistical approach^4,7,8^ to identify edges and nodes associated with the emergence of seizure precursors. First, we compared for each patient the distributions of values of the aforementioned qualifiers (mean phase coherence *R* for edges; centralities *C*^*B*^ and *C*^*S*^ for nodes) from an assumed pre-seizure (pre-ictal) period of ${T}_{{\rm{pre}}}=4\,{\rm{h}}$ duration with those from inter-ictal periods. We discarded data from the 30 min interval after the onset of a seizure (*T*_post_) to not bias our analyses with effects from the seizure and particularly from the post-ictal period (in cases where the time between two successive seizures was less than ${T}_{{\rm{pre}}}-30\,{\rm{\min }}$, the maximum amount of data available, i.e., from seizure onset back to the end of the post-ictal phase of the preceding seizure, was used instead).

For our investigations, we only considered clinical seizures with an inter-seizure interval that exceeded ${T}_{{\rm{pre}}}+{T}_{{\rm{post}}}$. For those seizures that met this inclusion criterion, the time of seizure onset was visually identified on the iEEG as the time of earliest clear change from the patient’s baseline or normal background iEEG that eventually led to an electrographic seizure. Subclinical seizures were neglected in our analyses.

We denote those nodes and edges as *predictive* if their pre-ictal and inter-ictal distributions of qualifiers differed significantly (Kolmogorov-Smirnov test; $p < 0.05$ after Bonferroni correction) and if the difference – taken as an estimate for prediction performance – exceeded chance level. The latter was evaluated by testing it against the null hypothesis of the non-existence of a pre-seizure state. For this purpose, we employed the concept of seizure time surrogates^7,69^ (19 seizure time surrogates; $p < 0.05$) that also allowed us to account for possible confounding influences such as seizure clustering, daily rhythms, and changes in anticonvulsive medication.

We then registered to which functional modules (or combinations thereof) these *predictive nodes* and *predictive edges* belonged to (Fig. 1). Finally, we checked, whether a module or module combination preferentially contained predictive edges or nodes, given the varying number of electrode contacts within each module or module combination (hypergeometric test; $p < 0.05$). We here only consider modules or module combinations that passed this test.

## Data Availability

The data that support the findings of this study are available from the corresponding author upon reasonable request. The data are not publicly available as they contain information that could compromise the privacy of research participants.
